# Renewable Resources as Promising Materials for Obtaining Graphene Oxide-like Structures

**DOI:** 10.3390/nano14191588

**Published:** 2024-10-01

**Authors:** Tilek Kuanyshbekov, Kydyrmolla Akatan, Nazim Guseinov, Renata Nemkaeva, Bayan Kurbanova, Zhandos Tolepov, Malika Tulegenova, Sana Kabdrakhmanova, Almira Zhilkashinova

**Affiliations:** 1 National Scientific Laboratory of Collective Use, Sarsen Amanzholov East Kazakhstan University, 55 Kazakhstan Str., Ust-Kamenogorsk 070002, Kazakhstan; ahnur.hj@mail.ru (K.A.); almira_1981@mail.ru (A.Z.); 2 Kaz Graphene, 63 Zapadnyi Str., Ust-Kamenogorsk 070011, Kazakhstan; solar_neo@mail.ru (N.G.); mr.tolepov@mail.ru (Z.T.); malika.tulegenova@bk.ru (M.T.); 3 Department of Physics, School of Sciences and Humanities, Nazarbayev University, Astana 010000, Kazakhstan; quasisensus@mail.ru; 4 Department of Physics and Technology, Al-Farabi Kazakh National University, Al-Farabi Avenue, 71, Almaty 050040, Kazakhstan; bayan.kurbanova@mail.ru; 5 Scientific Center of Composite Materials, 79 Nurmakovstr., Almaty 050026, Kazakhstan; sanaly33@mail.ru

**Keywords:** graphene oxide, carbon materials, activated carbon, coal, metamorphism, Hummers’ method

## Abstract

Currently, one of the topical directions in the field of production and application of graphene-like nanostructures is the use of renewable natural raw materials, which have unlimited resources for an economically efficient large-scale yield of a product with environmental safety. In this regard, we present the production of graphene oxide (GO) from a renewable natural raw material of plant biomass, birch activated carbon (BAC), and a comparison of the obtained physicochemical, mechanical, and electrical properties of birch activated carbon–graphene oxide (BAC–GO) and graphite–graphene oxide (G–GO) synthesized from the initial materials, BAC and graphite (G). Results obtained from this study confirm the successful oxidation of BAC, which correlates well with the physical–chemical dates of the G–GO and BAC–GO samples. Change in data after the oxidation of graphite and BAC was facilitated by the structure of the starting materials and, presumably, the location and content of functional oxygen-containing groups in the G–GO and BAC–GO chains. Based on the results, the application of a cost-effective, eco-friendly colloidal solution of nanodispersed BAC–GO from a plant biomass-based high-quality resource for producing large-scale nanostructured graphene is validated which has potential applicability in nanoelectronics, medicine, and other fields.

## 1. Introduction

The use of non-renewable petroleum products and synthetic materials is one of the main causes of human health issues and environmental problems [1]. Therefore, scientists of the world are keen on studying safe materials that can be obtained from renewable natural raw materials [2]. In this regard, results of research in recent years carried out by scientists working with plant biomass, namely nut coke [3,4], agricultural waste [5], and coconut shell [6], has resulted in widely used methods for the production of activated carbon materials for water purification [7], removal of soil organic pollutants [8], adsorption of toxic gases [9], as catalysts or catalyst supports [10], and as electrodes in supercapacitors [11]. In recent years, scientists have begun to focus on the issue of obtaining graphene oxide (GO) from activated carbon [12,13,14,15]. Graphene oxide (GO) is not only an intermediate product used in the production of graphene, but it also can be applied in its individual state in nanoelectronics, components of composite materials, solar cells, super capacitors, water purification membranes, adsorbents, quantum dots, and fluorescent materials for biology and medicine and has the possibility of being used as an activating agent of plant metabolism in agriculture [16,17,18,19]. 

Synthetic and natural graphite are widely used raw materials for the production of GO. Natural graphite oftentimes is not obtainable due to its low concentration in certain areas on the surface of the earth, and the raw material source of graphite is limited due to the rising demand for graphite in battery production in recent years. Synthetic graphite is very expensive because it is obtained from graphitized carbon as a by-product of oil at a temperature of 3000 °C [11,20]. Therefore, obtaining graphene oxide from alternative compliant, abundant, and low-cost material for large-scale production is a difficult task.

Among the carbonaceous materials, there is a similarity in the crystal structure of graphite and coal at a high stage of metamorphism (high-stage metamorphism) [21]. This can be explained by the rule that distance between aromatic layers in the hexagonal crystal lattice of graphite is d = 0.335 nm, the diameter is L_a_ = 20 nm, and the layer height is equal to L_c_ = 58 nm, and the average diameter of the aromatic layers in coal depends on their stage of metamorphism [21]. The average layer diameter of coal containing less than 75% carbon is equal to L_a_ ≈ 0.5 nm, which means that about 8–9 atoms are located in the aromatic layer. And if coal with a carbon share of 78–92% has a relatively stable layer diameter, L_a_ = 0.75 nm, corresponding to 15–18 atoms per layer, and if the carbon content is higher than 92%, the layer diameter increases sharply to L_a_ > 1 nm, L_c_ = 1.2–2.4 nm, and d = 0.346–0.355 nm. It means that the number of atoms in the aromatic layer exceeds 30 [22,23,24,25,26,27,28,29,30,31]. Research studies have shown that GO obtained via oxidizing natural coal resulted in the synthesis of a nanodispersed colloidal solution (rGO), and work was carried out to obtain solar cell electrodes [28], photocatalysts [29], and a carbon electrode for electrochemical determination of caffeine [30] as a component of a composite material. 

The typical characterization of a material’s crystalline structure often involves assessing parameters like the interplane distance d, as well as the dimensions of its structural components L^a^ and L^c^, alongside evaluating the degree of order. The dimensions of crystallites, L^c^, vary among different graphite samples and are influenced by the reflex chosen for calculation. The degree of ordering, interlayer spacing d^(002)^, and crystalline sizes (L^a^ and L^c^) are recognized as the primary parameters for assessing the stacking structure of carbonaceous materials [32].

L^a^ represents the average diameter of the crystallites in the basal plane, and L^c^ represents the average stack thickness perpendicular to the basal plane in perfectly coherent domains, i.e., crystallites. These are the main parameters for carbon characterization, which are usually calculated using the Scherrer equation through relevant peak widths, with X-ray diffraction patterns (XRD) commonly employed [33]. XRD also determines the lattice constants, a and c, which are typically derived indirectly using the Bragg equation. This is achieved by calculating the period of a family of atomic planes and relating it to the crystal system’s geometry [34]. Raman spectroscopy is a well-established, non-destructive technique used to acquire information on the bulk properties of polycrystalline materials [35,36,37,38], and it has increasingly been used to determine the crystallinity of carbon materials as an alternative to XRD [39].

In addition to Hummers’ method [40], reagents such as FeCl_3_, ZnCl_2_ [15], HCl, and HNO_3_ [11,29,30] and microwave [26] and hydrothermal treatment [31] methods were used to obtain rGO. However, the use of these methods increases the cost of the resulting product, as it requires a large amount of electricity. Also, the additional reagents used significantly increase the amount of liquid waste. Therefore, one of the ways to obtain economically efficient and safe production of GO is the use of renewable raw materials, i.e., plant biomass. 

Taking into account structural similarity between activated charcoal and graphite, it can be seen that it is a potential source of raw materials for synthesizing nanodispersed colloidal solutions of GO via strong oxidant structural breakdown and consequent introduction of oxygen-containing functional groups (epoxy, hydroxyl, and carboxyl) [41]. Waste generated during the processing of wood materials is an important source of raw materials in the production of activated carbon. 

By pyrolyzing birch (lat. Betula) wood waste in an airless environment and further processing it with water steam heated to 900 °C, activated carbon (birch activated carbon (BAC)) with the following parameters was obtained: average size of particles is 240 ± 20 mm with adsorption activity by iodine of 55 ± 10%, total pore volume of 1.6 cm^3^/g, apparent density of 240 g/dm^3^, area per unit volume of 600 ± 100 m^2^/g, and micropore volume of 0.28–0.33 cm^3^/g [42]. Since the raw material is available and renewable, the cost of the obtained coal can be considered cheap. Due to its high specific surface area and porosity, BAC can be used as a sorbent for the removal of heavy metals from water [43] and toxins from liver [44,45], or can be applied in the manufacture of supercapacitors [46,47], electrodes [46], and photocatalysts [48]. 

However, there are no studies on obtaining BAC–GO. Therefore, in this research work, nanodispersed colloidal GO solution was synthesized for the first time using Hummers’ method by oxidizing activated charcoal obtained from birch wood. Jointly, its physicochemical, mechanical, and electrical properties were compared with GO obtained from graphite.

## 2. Experimental

### 2.1. Materials

Potassium permanganate (KMnO_4_, 99%), sulfuric acid (98% H_2_SO_4_), hydrochloric acid (HCl, 99%), sodium nitrate (NaNO_3_, 99.5%), and hydrogen peroxide, (H_2_O_2_, 35%) were obtained from Sigma-Aldrich (Bangalore, India). BAC was obtained from Alita LLP (Kazakhstan). All other reagents were of analytical grade and were used without additional purification.

### 2.2. Methods

#### 2.2.1. Preparation of GO from BAC and Graphite 

**Figure 1:**
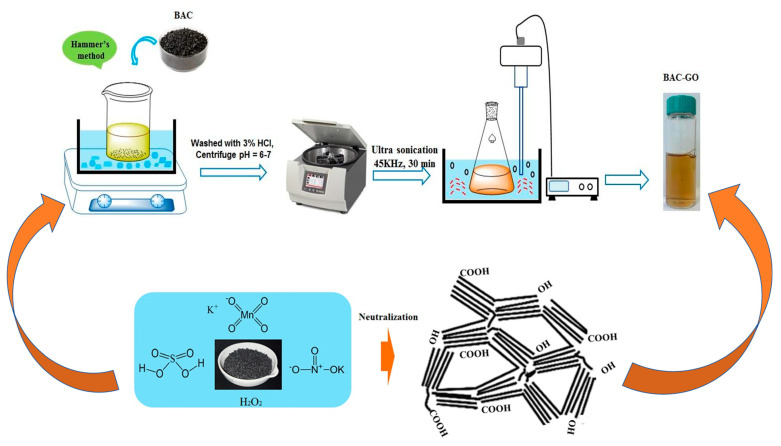
Scheme representing the preparation of GO.

GO is synthesized from activated carbon via Hummers’ method (Figure 1). Obtaining GO has the following sequence: the flask is filled with 6 g of activated carbon, which is placed in an ice bath with a temperature of 0 °C and stirred continuously for 15 min, while adding 23 mL of 94% H_2_SO_4_ via dripping. To the resulting suspension, 3 g of NaNO_3_ is added; then, while stirring the mixture, 18 g of KMnO_4_ is gradually added while maintaining the temperature below 20 °C for 2 h; afterwards, the temperature is raised to and maintained at 35 °C for 30 min. Then, after adding the deionized water, the temperature of the mixture rose to 90 °C. Finally, 30% H_2_O_2_ is added until the mixture changes color to light yellow. 

At the end of the reaction, the product is centrifuged and washed several times in 3% HCl solution to remove residual metal ions and neutralized with deionized water until the pH = 6–7. Finally, the suspension was sonicated at a frequency of 45 kHz for 30 min. The extraction process of GO from graphite was carried out according to the above methodology. The synthesis of GO from graphite was carried out according to the above methodology.

#### 2.2.2. Ultraviolet–Visible Spectroscopy (UV-Vis) Analysis of B–GO and G–GO

The optical absorption spectrum of the obtained BAC–GO and graphite–graphene oxide (G–GO) aqueous solutions were recorded on a spectrophotometer (PE-5400UV, Ecroskhim, Russian) with a scanning speed of 240 nm/min and a wavelength of 190–1000 nm. A 10 mm thick quartz cuvette was used.

#### 2.2.3. Fourier Transform Infrared (FTIR) Spectroscopy

FTIR analysis of BAC–GO and G–GO were performed on an FT-801 FTIR spectrometer (Simex, Russia) with a resolution of 1 cm^−1^ at a range of 450–4700 cm^−1^, in accordance with the standard method and using an accessory to measure attenuated total reflection (ATR) and specular-diffuse reflection (SDR) at a temperature of 25 ± 1 °C. 

#### 2.2.4. Raman Spectroscopy

To study samples of graphite, G–GO, BAC, and BAC–GO, a combined system of a Raman spectrometer Solver Spectrum (NT-MDT, Russia) was used. This study was carried out with the following parameters: laser 473 nm, grating 600/600, and signal accumulation time 30 s.

#### 2.2.5. Determination of Functional Oxygen-Containing Groups in the BAC–GO and G–GO Samples

The amount of oxygen groups in the raw materials, BAC and graphite, and the synthesized BAC–GO and G–GO was comparatively determined via Boehm’s method [49]. In the first place, a sample of 0.250 g of the test material was placed in a weighed conical flask with a capacity of 50 mL. Then, 25 mL of 0.1 M sodium hydroxide solution was added to the sample. The mixture was put on a magnetic stirrer for 30 min and filtered through a dense filter paper for fine precipitates. Three samples of 5 mL each were taken from the filtrate, transferred to a 50 mL conical flask, and titrated with 0.1 M HCl solution. The content of N groups was calculated with the following formula, [(mg-eq.)/g]:

N = (a − b)⋅0.1·5/m (1)

where N is the total number of acidic groups in the oxidized carbon material; a is the volume of 0.1 M HCl solution used for titration of the control sample, mL; b is the amount of 0.1 M HCl solution used for titration of the analyzed sample (filtrate), mL; 25 is the volume of 0.1 M analyzed solution taken for processing, mL; 5 is the volume of the filtrate taken for titration, mL; m is the sample weight, g.

#### 2.2.6. The Particle Sizes of BAC–GO and G–GO

A 1 wt% aqueous suspension of BAC–GO and G–GO were subjected to 30 kHz for 10 min using an ultrasonic disperser, U-sonic UZTA-0.15/22-0 (Alena, Russia). The average particle size was determined via dynamic light scattering (DLS) using a Zetasizer NanoZS 90 (Malvern, UK).

#### 2.2.7. X-ray Photoelectron Spectroscopy (XPS) Analysis 

For XPS analysis, graphite, G–GO, BAC, and BAC–GO samples were drop cast onto silicon (Si) substrates. The measurements were carried out on the equipment Nexsa G2 Surface Analysis System (Nexsa Analytical Ltd., UK.) with a monochromatic X-ray source Al Ka (hv = 1486.81 eV) (36 W, spot diameter 400 μm). Narrow scans (C1s: 280–298 eV) were performed at a passage energy (CAE) of 50 eV and a step size of 0.1 eV. The narrow scan spectra were shifted to the main carbon peak at 284.8 eV.

#### 2.2.8. X-ray Diffraction (XRD) Analysis 

The crystal structures of graphite, G–GO, BAC, and BAC–GO were studied via X-ray diffraction on a X’PertPRO diffractometer (Malvern Panalytical Empyrean, The Netherlands) using monochromatized copper (CuKα) at a scan speed of 0.05° for 10 s, with a K-Alpha1 wavelength of 1.54187 Å. Measurement in reflection mode, using an aluminium rectangular multi-purpose sample holder (PW1172/01), was performed at a diffraction angle 2θ between 3° and 40°, with the X-ray tube voltage was set at 45 kV, the current intensity was 30 mA, and the measurement time of each step was 0.5 s. The ICDD PDF-4/AXIOM database of XRD patterns was used for the analysis of the resultant XRD patterns. 

The graphene periodicity was determined via the following Scherer formula:

D = kλ/βcosθ(2)

where k is a shape factor that is often 0.94 [50]; λ is the wavelength of the diffractometer (0.1542 nm); β is the FWHM (maximum half of the full width) of the diffraction peak in radians; and θ is the diffraction angle.

#### 2.2.9. Scanning Electron Microscopy (SEM) with Energy-Dispersive X-ray Spectroscopy (EDX) Analysis 

Surface morphology and EDX analysis of the samples of graphite, G–GO, BAC, and BAC–GO were studied using SEM equipped with an EDX analysis system (SEM Quanta 200i 3D) (FEITM, Netherlands). The measurements were carried out in high-vacuum mode using a secondary electron detector at an accelerating voltage of 15 kV.

#### 2.2.10. Brillouin Spectra 

Brillouin spectra of graphite, G–GO, BAC, and BAC–GO were recorded in the 180°-backscattering geometry using a (3+3)-pass tandem JRS Fabry-Perot Interferometer [51] (JRS Scientific Instruments, Switzerland). The free spectral range was set at 25 GHz. The incident 532 nm wavelength laser light was focused down to 2 µm spot diameter using 20× microscope objective, while the optical power of the laser beam was kept below 10 mW to prevent sample damage.

#### 2.2.11. Density, Sound Velocity, and Young Modulus 

Ultrasonic velocity and density measurements of graphite, G–GO, BAC, and BAC–GO were performed using an Anton Paar World Scientific density and sound velocity meter (DSA 5000 M) with an accuracy of ±0.5 m/min and ±0.000005 g/cm^3^, respectively. The instrument was fully automated, and the temperature was controlled automatically. 

#### 2.2.12. Electrical Characteristics

To obtain the electrical properties of the graphite, G–GO, BAC, and BAC–GO samples, electrical measurements were carried out using the traditional four-probe method, with the current measured using a Keithley 648kei5 (Tektronix, USA) and voltage applied using a Tektronix PWS2326. 

#### 2.2.13. Adsorption Porosimetry

The textural properties of G–GO and BAC–GO were evaluated by employing adsorption porosimetry, specifically low-temperature nitrogen adsorption. This analysis was conducted using an Autosorb 1 surface area and porosity analyzer (Quantachrome Instruments, USA) and an ASAP-2020 adsorption analyzer (Micromeritics, USA). Before analysis, the samples with an average mass of 77.1 mg were degassed at a temperature of 115 °C for 2 h. BET analysis was carried out for a relative vapor pressure of 0–1 at −196 °C (77.3 K).

#### 2.2.14. Creation of a Humidity Sensor Based on BAC–GO and G–GO

**Figure 2:**
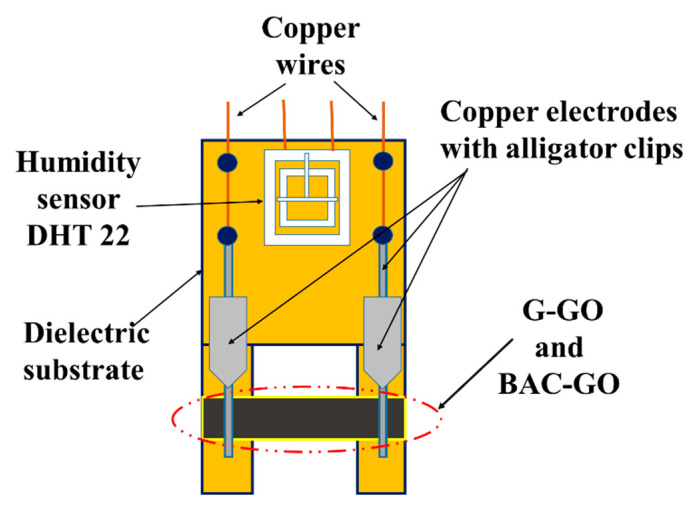
Structure of the humidity sensor.

**Figure 3:**
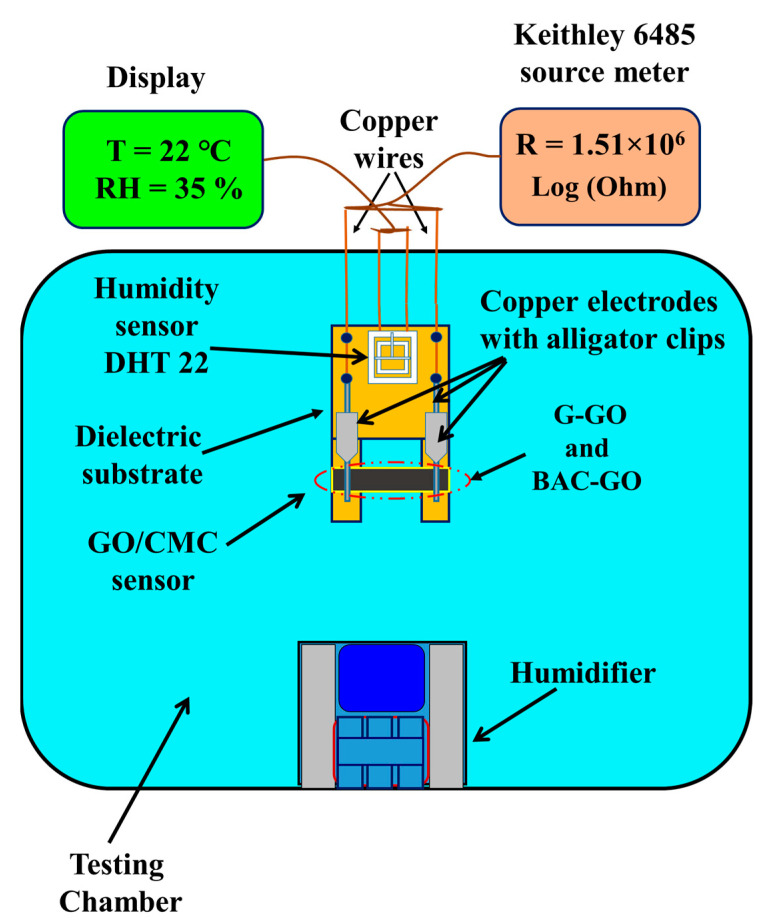
Schematic view of the installation.

The design of the humidity sensor is shown in Figure 2. The BAC–GO and G–GO membranes were mounted on a dielectric substrate and connected with opposite ends of copper wire (0.15 mm in diameter) as electrodes to the samples. These electrodes were attached with stable fixtures to ensure good electrical contact. Four legs of the dielectric substrate were installed. As can be seen from Figure 2, the sensor substrate had the following dimensions: 10.5 cm × 5.7 cm; the thickness of the membranes was about 20 microns; the length was 3.5 cm; and the width was 1 cm. The surface of the BAC–GO and G–GO membranes was open on both sides, making it sensitive to the relative humidity of the environment. As can be seen from the diagram in Figure 2, a humidity sensor based on BAC–GO and G–GO and an exemplary DHT 22 sensor on the Arduino platform were mounted together on a dielectric substrate and placed together in the test chamber (Figure 3) that was used to control the humidity. The DHT 22 sensor is designed to measure humidity levels in the range from 0 to 100% and the measurement accuracy is in the range of 2–5%. 

A schematic representation of the installation for investigating the sensitivity of the sensor to humidity is shown in Figure 3. The humidity sensor test chamber has a main box and a humidity controller from 1% to 99% with a temperature sensor. A Keithley Picoammeter (Picoammeter 6485× Model, Tektronix, USA) was used to measure the electrophysical characteristics of the humidity sensor. The Keithley 6485 m was attached to the copper electrodes with alligator clips. The error bars were limited by an instrument error of no more than 0.5%.

## 3. Results and Discussion

### 3.1. UV-Vis Analysis of BAC–GO and G–GO

**Figure 4:**
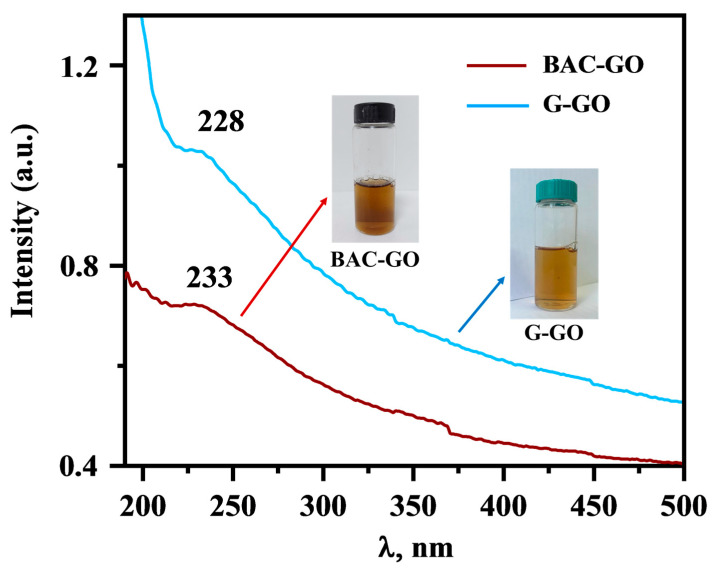
UV spectra of G–GO and BAC–GO suspension.

Figure 4 shows the relative UV spectrum of BAC- and graphite-derived GO. In the spectrum of BAC–GO, an absorption band was observed at a wavelength of 228 nm, and for G–GO, a wavelength of 233 nm was observed. This indicates the presence of a π-π* bond of the aromatic ring in the GO molecule and C=O bonds in the epoxy; carbonyl groups formed after strong oxidation [19,20] (Table 1). The obtained results are in good agreement with other studies [52,53,54] in which absorption was found in the 220–240 nm region. 

According to the obtained UV spectra, it is found that GO from graphite and BAC are similar, with only a noticeable difference in their intensities.

### 3.2. FTIR Spectroscopy 

**Figure 5:**
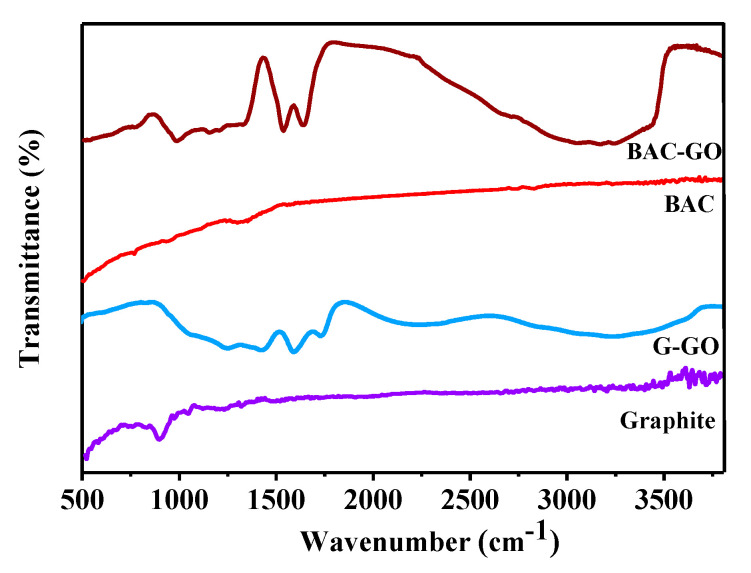
FTIR spectra of graphite, G–GO, BAC, and BAC–GO.

Figure 5 shows the comparative FTIR spectra of the starting materials and the studied GO. Weak signals of C-O-H and C=O bonds were observed in the spectra of BAC–GO and G–GO at wavenumbers 980 cm^−1^ and 1417 cm^−1^, respectively (Table 1) [55]. This was further clarified by the accuracy of the numerical values of oxygen groups (0.2 mg-eq/g) in BAC and graphite molecules in Table 2. And, according to the obtained spectra of BAC–GO and G–GO, the absorption regions at 3300 cm^−1^ and 1420 cm^−1^ are due to O-H, 1723 cm^−1^ represents the long vibration of C=O bonds in the carbonyl group and carboxyl groups, long-term vibrations and deformation peaks of C=C bonds of the aromatic ring are present at 1585 cm^−1^, absorption at 1249 cm^−1^ is attributed to long-range vibration of C–O epoxy functional groups, and 1054 cm^−1^ was found to be characteristic of long-range vibrations of C–O alkoxy bonds [54,55,56]. In the course of strong oxidation, we can see that intense absorption peaks of oxygenated hydroxyl, epoxy, carboxyl, and carbonyl groups connected with carbon atoms appear. From this, we can see that the increase in the numerical values of the bound oxygen groups in Table 2 are on the order of 65 times in BAC–GO and 75 times in G–GO, which is clearly observed in the FTIR spectrum. Similar research results were obtained in other studies [13,23,40,56].

Based on this, the FTIR spectra data confirm that the BAC derived from renewable plant biomass and the GO synthesized from it are of high quality and are similar to the GO obtained from synthetic graphite. The synthesis efficiency (yield) of GO from synthetic graphite is 10.4%, and that of GO from BAC is 11.6%, nearly 1.2% higher than the yield of GO from synthetic graphite. In this regard, based on the above data, in comparison with synthetic graphite, BAC is the more efficient and economical material for the synthesis of BAC–GO. According to IR spectra in the works [50,51,52,57,58] on the production of graphene-like nanomaterials from various biodegradable wastes and biomass, the presence of functional oxygen-containing groups was detected in the wavenumber range from 950 to 3500 cm^−1^, which is in good agreement with our results.

### 3.3. Raman Spectroscopy

**Figure 6:**
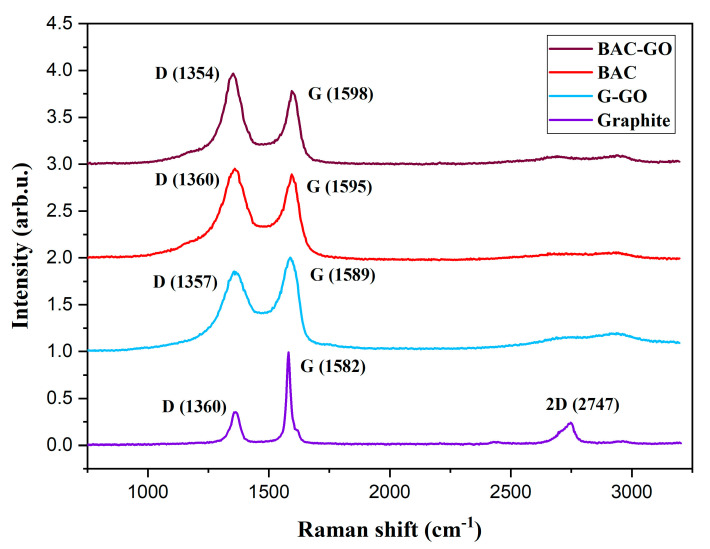
Raman spectra of graphite, G–GO, BAC, and BAC–GO.

Raman spectroscopy is a fundamental method for studying characteristics of carbon nanostructures, the transverse size of crystals, the density of defects, and the quality of the graphene layer and is used to describe the quality of graphene in manufacturing processes since it is not destructive [59,60,61,62,63,64,65,66,67,68,69,70,71]. Basically, the Raman spectra of carbon and carbon nanomaterials are characterized by two main peaks, designated as G and D peaks near 1589 and 1351 cm^−1^. The G peak originates from stretching of sp^2^ bonded carbon, while the D peak is due to the breathing mode of the C6 hexagonal ring, typically observed in disordered sp^2^-bonded carbon. In the case of graphite and few-layered graphene nanostructures, the Raman spectra present three main peaks, D, G, and a 2D peak. The 2D peak around 2730 cm^−1^ represents the second order of the D band; however, unlike the D band, the 2D peak does not require the presence of defects to be observed in graphitic materials. Increase in FWHM and a decrease in intensity of the 2D peak indicate the high level of disorder in sp^2^ graphitic structures. Figure 6 shows the Raman results of our graphite, G–GO, BAC, and BAC–GO samples, and, according to the shown spectra, there are distinctive features in the peak positions and shapes of the G, D, and 2D peaks, and the relative intensity (which are normalized from 0 to 1) of these peaks also varies significantly. According to the Raman spectra in graphite, G–GO, BAC, and BAC–GO samples, there is a G peak due to in-plane stretching between sp^2^ carbon atoms and a D band recognized as a disordered band due to structural defects, edge effects and dangling sp2 carbon bonds that break the symmetry [11,59,60,61,62,63,64,65,66,67,68,69,70,71,72,73,74,75,76,77,78,79,80]. 

For the initial graphite, the positions of the D and G peaks are in the center of 1360 cm^−1^ and 1582 cm^−1^; the 2D peak is at 2747 cm^−1^ (Table 3, Figure 6). After its oxidation via the Hummers’ method, an increase in the intensities of the D peak (from 0.36 to 0.88) and a position shift (from 1360 to 1357 cm^−1^) are observed, and the G peak is broadened. However, the intensity remains almost unchanged; however, a shift in the position of the G band towards higher wave numbers (from 1582 to 1589 cm^−1^) is more noticeable in G–GO than in the graphite sample. The increase in the intensities of the D peak is explained by the introduction of oxygen functional groups into the graphite chain [11,59,60,61,62,63,64,65,66,67,68,69,70,71]. Accordingly, the number of defects on the carbon surfaces increases, which contributes to a greater increase in the I_D_/I_G_ of the G–GO sample by almost 2.5 times (from 0.36 to 0.88) compared to that of graphite (Table 3, Figure 6). A change in position of the G band towards higher wave numbers during graphite amorphization indicates the presence of double bonds that resonate with higher wave numbers, which confirms the successful oxidation of graphite via the Hummers’ method [11,13,23,40,56,59,60,61,62,63,64,65,66,67,68,69,70,71,72,73,81,82]. The shift of the G band from 1582 to 1589 in the G–GO sample, as well as the change in the intensity ratios of the D and G bands (I_D_/I_G_) from 0.36 to 0.88, approximately agrees with the data obtained in [11,59,60,61,62,63,64,65,66,67,68,69,70,71], where they vary in the range from 1575 to 1595 cm^−1^. 

The same distinctive changes in the shape and position of the Raman peaks are observed between the initial BAC and BAC–GO samples. The D peak of the initial BAC is located at 1360 cm^−1^ with intensity (I_D_ = 1) and the G peak is at 1595 cm^−1^ (I_G_ = 0.8). For BAC–GO, these peaks are centered at 1354 cm^−1^ (I_D_ = 1) and 1598 (I_G_ = 0.7) cm^−1^, respectively (Figure 4). After BAC oxidation, a noticeable difference is observed in the positions and intensities of the D and G peaks, with the D band shifting to lower wave numbers (from 1360 to 1354 cm^−1^). The slight increase in the intensity of the D peak compared to the initial BAC is due to the introduction of oxygen functional groups, primarily located at the edges, or within the pores of the tunnel-shaped BAC–GO honeycomb structure (Figure 6) [11,13,21,23,30,40,56,74,75,76,77,78,79,80,83]. The G peak shows low intensity relative to the D peak in both the initial BAC and BAC–GO, which is explained by the fact that BAC inherently has a higher disordered structure than graphite and G–GO [72,73,81,82,84,85,86,87]. Compared to BAC, there is a shift of the G band in the BAC–GO sample towards higher wave numbers. This is due to the increase in disorder in the graphitic structure and a rise in additional defect modes, leading to the widening of this band during BAC amorphization, which agrees with the G–GO data and confirms the successful oxidation of BAC via the Hummers’ method [11,13,23,40,55,59,60,61,62,63,64,65,66,67,68,69,70,71,72,73,81,82]. In addition, the successful modification of BAC is indicated by the increase in the intensity ratio of the D and G bands (I_D_/I_G_) from 1.23 to 1.38, approximately 1.12-times higher compared to the initial BAC. This result is consistent with other studies [11,59,60,61,62,63,64,65,66,67,68,69,70,71], which observe D and G peak shifts in the region from 1345 to 1357, and from 1585 to 1604, respectively, with I_D_/I_G_ ratios ranging from 1.00 to 2.21. 

The most significant results of Raman scattering are the determination of the crystal size of the initial samples of graphite from BAC before and after their oxidation via the Hummers’ method, according to the following Tuinstra Koenig relation [35]:

L_a_ (nm) = (2.4 × 10^−10^) × λ^4^ (I_D_/I_G_)^−1^ (3)

where λ if the laser wavelength (nm), and I_G_ and I_D_ are intensities of the G and D peaks, respectively.

According to Table 3, a change in the crystal size is observed; compared with the initial samples of graphite and BAC, after their oxidation using the Hummers’ method, the L_a_ values of graphite and G–GO samples decreased by almost 2.4 times from 33.09 nm to 13.69 nm, and in BAC, BAC–GO decreased by 1.13 times from 9.8 to 8.67. The decrease in the crystal size of G–GO depends on the structure and shape of the initial material. Since graphite crystals are mainly flaky, scaly, or lamellar, respectively, after Hummers’ method, 3D bulk graphite is able to split into multilayer and even single-layer graphene sheets, which in turn affects the size reduction [11,74,75,76,77,78,79,80]. Compared to G–GO, the BAC–GO sample showed the smallest decrease in values, since the initial BAC is a highly developed porous rod-like structure, which affected the insignificant change in the crystal size. The L_a_ values of the graphite, G–GO, BAC, and BAC–GO are approximately similar to the data in [11,59,60,61,62,63,64,65,66,67,68,69,70,71], where they vary in the range from about 18 nm to 120 nm. 

**Figure 7:**
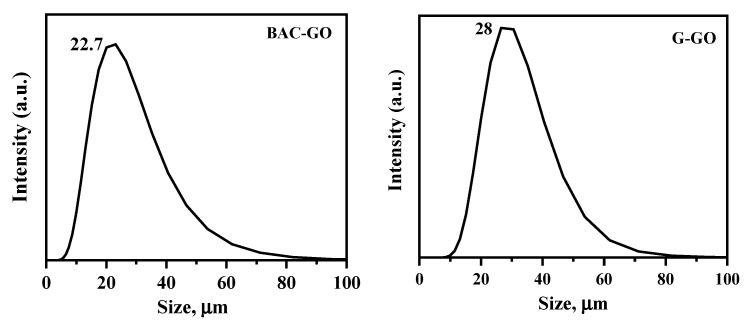
The particle sizes of graphite, G–GO, BAC, and BAC–GO.

**Figure 8:**
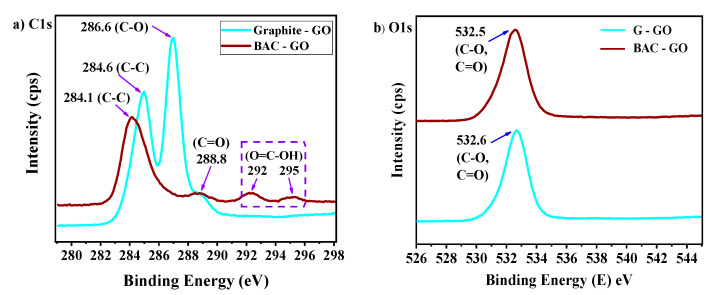
XPS spectra of (**a**) C1s and (**b**) O1s for G–GO and BAC–GO.

**Figure 9:**
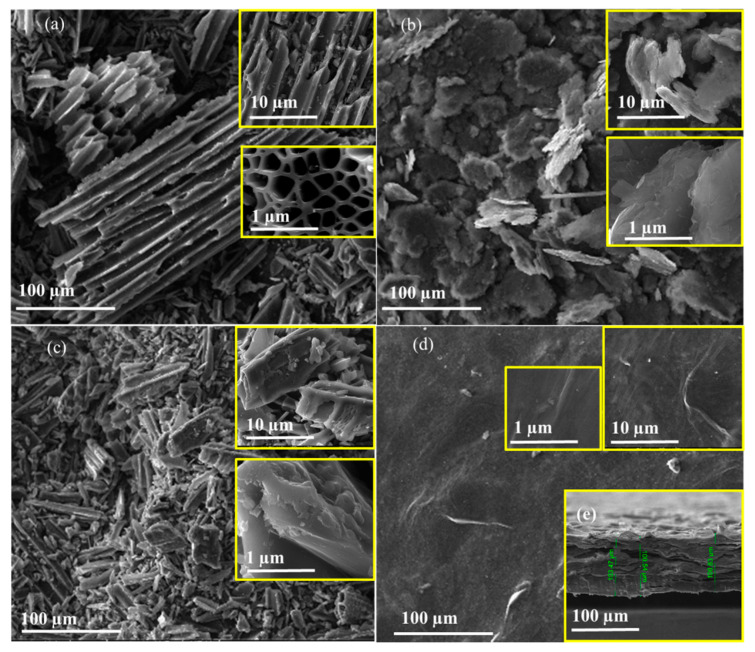
SEM images of (**a**) BAC, (**b**) graphite, (**c**) BAC–GO, (**d**) G–GO, and (**e**) cross section of G–GO.

**Figure 10:**
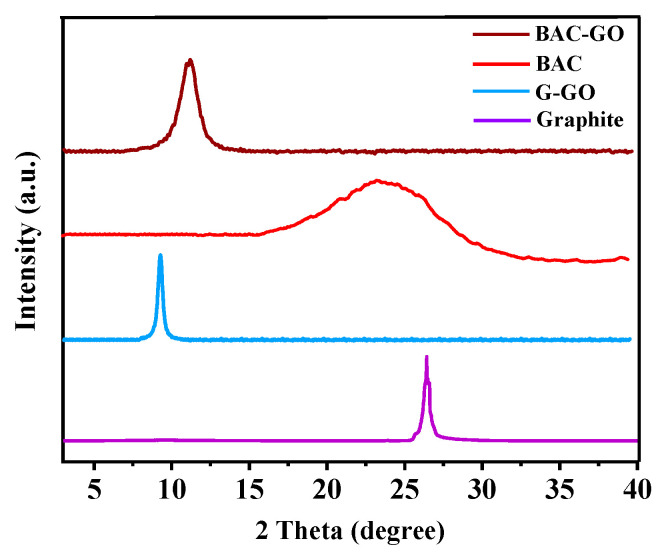
XRD patterns of graphite, G–GO, BAC, and BAC–GO.

**Figure 11:**
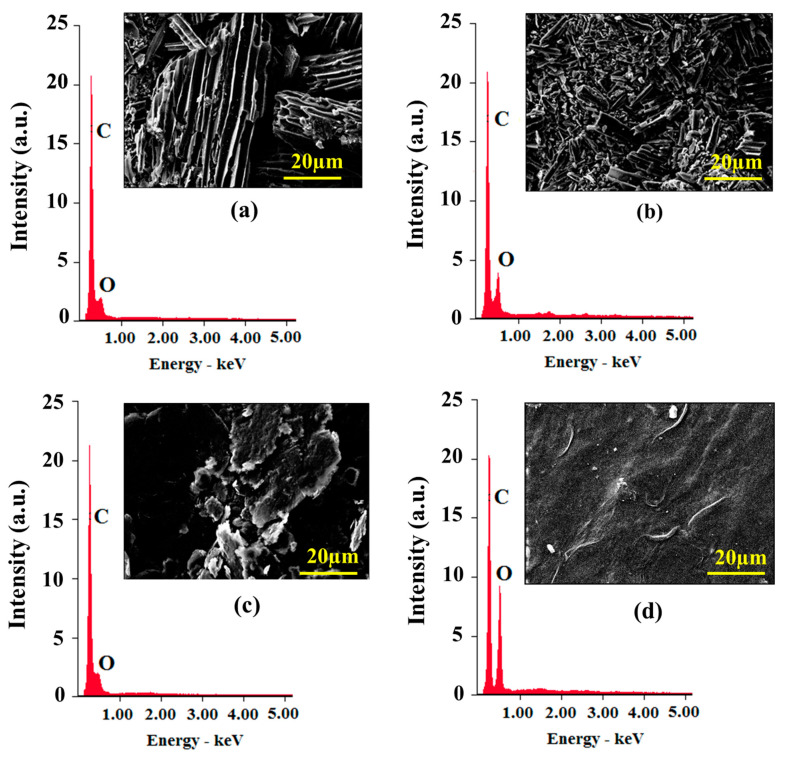
EDX analysis of (**a**) BAC, (**b**) BAC–GO, (**c**) graphite, and (**d**) G–GO.

In general, according to the obtained results of Raman spectroscopy (Table 3, Figure 6), in the comparison of G–GO and BAC–GO, depending on the initial samples of graphite and BAC, changes are clearly observed in the positions, intensities, and shapes of the peaks, which in turn is in good agreement with the known literature data [11,59,60,61,62,63,64,65,66,67,68,69,70,71]. Moreover, a good correlation between UV, XPS, IR, Raman, XRD, SEM, EDX (Figures 4–11), and determination of the level of oxygen-containing groups (Table 2), respectively, confirms the successful oxidation of graphite and BAC using the Hummers’ method. The most important point in the successful synthesis of G–GO and BAC–GO is the measurement of the intensity ratio of the D and G bands (I_D_/I_G_) and the determination of the level of functional oxygen-containing groups (Table 2). The incorporation of oxygen functional groups in the G–GO was more than 2.4 times greater than the initial graphite. Similar changes were observed in the ratio of the intensities of the D and G bands (I_D_/I_G_) of the BAC and BAC–GO samples, which increased by almost 1.13 times from that of the initial BAC, respectively, which is in good agreement with the determination of the level of oxygen-containing groups (Table 2). A distinctive difference is that the level of oxygen-containing groups and the I_D_/I_G_ ratio of the BAC–GO sample turned out to be lower than those of G–GO, since in G–GO, the functional oxygen-containing groups were attached to the basal plane and along the edges [11,21,54,55,56,59,88,89,90,91,92,93,94,95]. While in BAC–GO, due to a structural feature, these groups are located at the edges, or they may have penetrated into the pores of the tunnel-shaped, rod-shaped common honeycomb structure of BAC–GO [11,59,60,61,62,63,64,65,66,67,68,69,70,71,74,75,76,77,78,79,80,83]. According to the known literature Raman spectra data [42,43,44], the D peaks were detected in the region of 1300–1400 cm^−1^, and G peaks were detected from 1500 to 1600 cm^−1^, coinciding well with our results and confirming the successful preparation of G–GO and BAC–GO. 

### 3.4. The Particle Sizes and Functional Oxygen-Containing Groups in the BAC–GO and G–GO Samples

Figure 7 shows the average particle sizes in the obtained BAC–GO and G–GO aqueous suspensions. The average particle size of BAC–GO was 22.7 ± 2 μm, and G–GO was 28 ± 2 μm. The size of GO obtained from BAC was found to be about 5 μm smaller than GO obtained from graphite.

In Table 2, comparing the raw material and the obtained GO, we see that the difference in numerical values is large. It turned out that in the initial BAC, the content of oxygen-containing groups was 0.2 mg-eq/g; after strong oxidation, the content of oxygen-containing groups in the obtained BAC–GO increased to 13 mg-eq/g. This was 65-times more than the initial BAC. And in initial graphite and synthesized G–GO an increase of 75 times was observed, from 0.2 mg-eq/g to 15 mg-eq/g.

It was found that the amount of bound oxygen groups (oxygen-containing groups) in the GO molecule obtained from this BAC is less than 2 mg-eq/g compared to GO obtained from graphite with an ordered crystal structure that intensively affects the attraction of oxygen atoms. In the study [56], the amount of oxygen-containing groups in the GO molecule obtained via surface modification of coal was 6.6 mg-eq/g, which was 16.5-times lower than that of the initial raw material. Moreover, the result obtained in this study is 2-times higher than the result of [56].

### 3.5. XPS Analysis

To determine the surface functional oxygen-containing groups of GO from graphite and BAC, we studied the distribution of each component of the C1s and O1s peaks, which were obtained from the deconvolution of the spectra of the high-resolution C1s and O1s nucleuses (Figure 8a,b). According to the results for C1s spectra via XPS analysis of GO obtained from graphite, main peaks were found with binding energies of 284.6; 286.6; and 288.8 eV associated with sp^2^ aromatic carbon atoms (C=C), epoxy groups (C-O-C), and carboxyl groups (C(O)-O-H), respectively (Figure 8a, Table 4).

The C1s peak positions of the G–GO sample at 284.6; 286.6; and 288.8 eV are in good agreement with the known data in [11,14,72,73,81,82,84,85,86,87,96,97,98], where their peak positions are mainly displayed at 284.6, 286.6, 288.0, and 289.5 eV, which indicates that the oxidation of graphite using the Hummers’ method was successful.

The second sample shows covalently bonded carbon existing in BAC–GO as sp^2^ carbon (C-C/C=C, 284.1 eV), carbonyl/carboxyl groups (C=O/O-C=O, 288.8 eV), and peaks in the region of 292–295 eV, which are characteristic of the binding energies of π-π* transitions in aromatic closed chains (Figure 8, Table 4). These BAC–GO assignments are in good agreement with the values reported in the literature [11,99,100], where they show these peaks at approximately 284.4 eV (C=C, sp^2^ unoxidized graphitic carbon) and 288.8 eV (O-C=O, carboxyl), which proves the successful oxidation of BAC using the Hummers’ method.

According to the obtained C1s and O1s regions of the XPS spectra, it is clearly seen that, compared to BAC–GO, G–GO has a higher peak intensity, as well as a higher binding energy of 284.6 eV and 532.6 eV, than that of BAC–GO at 284.1 eV and 532.5 eV (Figure 8a,b). The second G–GO peak, characteristic of epoxy groups (-C-O-C-) with a binding energy of 286.6 eV, also has a high intensity. However, this peak was not observed in BAC–GO. Instead, the peak corresponding to carboxyl groups, with a binding energy of 288.8 eV, is in good agreement. In addition, compared to G–GO, in the XPS spectra of BAC–GO, a distinctive feature with the appearance of peaks with binding energies in the region of 292 and 295 eV was observed, which is characteristic of bonds of π–π* transitions in aromatic closed chains (Table 4) [11,72,99,100].

The corresponding narrow-scan photoemission spectra for oxygen (O1s) of G–GO and BAC–GO show contributions from singly and doubly bonded oxygen to carbon at 532.6 and 532.5 eV (Figure 8b), respectively, due to C–O and C=O groups, such as ketone, carbonyl, hydroxyl, and epoxy functionalities. The change in peak intensities and oxidation was influenced by the shape of the initial materials; graphite is a flake, and BAC has a rod-like shape, as a result of which, when GO is obtained from graphite, GO will have a layered structure, and BAC–GO will have a fibrous rod-like structure (Figure 9) [74,75,76,77,78,79,80,83].

According to the known literature data [11,14,50,59,60,61,62,63,64,65,66,67,68,69,70,71,72,73,74,75,76,77,78,79,80,81,82,83,84,85,86,87,96,97,98,99,100], the distinctive features of the structures of the initial materials contribute to the arrangement of functional oxygen-containing groups. Respectively, oxygen-containing functional groups sit well on the basal plane and along the edges of graphite GO due to the flake-like shape, and in BAC–GO, oxygen-containing groups are attached mainly along the periphery, which will affect the location of the intensities and bond energies of the obtained spectra from XPS analysis. Thus, results showed that graphite and BAC were successfully synthesized using the Hummers’ method, resulting in the appearance of functional oxygen-containing groups, which were confirmed via the obtained XPS spectra and are also in good agreement with SEM, UV spectra, Raman spectroscopy, IR, XRD, EDX (Figures 4–11), and determination of the level of oxygen-containing groups (Table 2) [11,14,60,61,62,63,64,65,66,67,68,69,70,71,96]. According to the indicated results, it was determined that the binding energy of atoms in the GO molecule obtained from two raw materials is in the range of 284–296 Ev, which indicated that GO was successfully obtained from BAC. According to the known literature data [52,53,54], the XPS spectra of the C1s binding energy were detected in the region from 283 to 290 Ev, which is in good agreement with our results.

### 3.6. XRD

Features of the crystal structures of the initial carbon material and those obtained after strong oxidation were studied (Figure 10; Table 5 and Table 6). In the diffractogram of the raw material BAC, 2θ = 23.46° (002), and the inter-layer distance was equal to d = 3.59 Å. This is characteristic of the structure of carbon with saturated (amorphous) aliphatic groups connected to carbon (graphite) crystallites via side chains [11,91]. The authors of [48,57] developed a similar concept as a result of X-ray diffraction analysis. Moreover, in graphite, 2θ = 26.43° (002), and the inter-layer distance d=3.5 Å. It was determined that this corresponds to the crystal structure of typical graphite [26,94]. Regarding the crystal structure of BAC–GO, it was found that the diffraction peak characteristic of GO was observed in the fracture zone 2θ = 11.91° (002), and the inter-layer distance was d = 8.2 Å, which is 2.28-times larger than the initial (d = 3.59 Å). It was found that the diffraction peak of G–GO is 2θ = 7.05° (002), and the inter-layer distance d= 10.2 Å, which is 2.9-times higher compared to the initial graphite. This is explained by the effect of epoxy, carbonyl, and hydroxyl oxygen functional groups incorporated into the GO molecule during strong oxidation in the synthesis [11,21,30,40]. This shows that the FTIR result (Figure 5) is accurate. The results of [13,92,95] are for GO obtained from natural coal, which has a similar crystalline structure. From this, it was shown that the carbon material obtained in this study is confirmed as GO according to its crystal structure. The XRD results we obtained are consistent with the previous work [52,53,54] on the production of graphene-like materials from biodegradable waste and biomass. Peaks at 2θ were detected in the range of 7° to 15°, confirming our successful production of BAC–GO and G–GO.

### 3.7. SEM Analysis 

We obtained SEM images of the starting materials of graphite and BAC and obtained samples of BAC–GO and G–GO from them after synthesis via the Hummers’ method. According to the images, one can clearly see the distinctive morphological and structural features of the obtained BAC–GO and G–GO samples depending on their initial materials, BAC and graphite (Figure 9). By their nature, graphite crystals are mainly flaky, scaly, or lamellar in shape, and activated carbon is a highly developed porous, rod-like structure. Also, in BAC, the orientation of individual lattice planes relative to each other, which is typical for graphite, is violated: the layers are randomly shifted and do not coincide in the direction perpendicular to their plane [50,74,75,76,77,78,98,99,100]. Figure 9a shows that the carbon surface is composed of tunnel-shaped pores and a common honeycomb structure. After synthesizing BAC via the Hummers’ method, it is clearly seen that the cellular holes of the activated carbon were fully developed, and the angular lines of the holes could be clearly observed (Figure 9c).

The surface morphology of BAC–GO shows that BAC before modification had numerous tunnel-shaped pores, and after functionalization, it is obviously observed that the surface became rougher. This is due to the attachment of numerous functional oxygen-containing groups, where they are in good agreement with the results of UV, XPS, IR, Raman, XRD, SEM, EDX (Figures 4–11), and oxygen-containing groups (Table 2). It is also presumably observed that the functional oxygen-containing groups have penetrated into the tunnel-shaped pores of BAC–GO, and the surfaces appear more distinct (Figure 9c) [11,59,60,61,62,63,64,65,66,67,68,69,70,71,74,75,76,77,78,79,80,83]. In addition, in the images of BAC–GO in Figure 9a,c, different pores of different diameters can be observed which are characteristically large pores in the range from 20 to 190 μm. According to the literature [85,86], according to the pore size, activated carbon can be divided into micropores (<2 nm), mesopores (2–50 nm), and macropores (>50 nm), and in our case, for the BAC–GO sample obtained by us, the pore sizes refer to materials having macropores. In addition, the SEM images of the obtained BAC–GO in Figure 9c show that the orientation of the particles relative to each other is disturbed and the layers are randomly shifted, which in turn contributes to the formation of the BAC–GO membrane. However, the G–GO sample shows a layered membrane in Figure 9d due to the scaly-lamellar shape and the consistent orientation of the particles from the initial graphite, as seen in Figure 9b [74,75,76,77,78,79,80,83]. According to the cross-sectional image (Figure 9e) the thickness of the G–GO membrane is about ~100 µm, and it is clearly seen that it has a layered sandwich-like, densely packed structure.

After graphite oxidization, the SEM image of the G–GO surface differs in that the surface became smoother, and wrinkled areas were found in places due to the scaly shape of the initial material (Figure 9d). Compared to BAC–GO, it is evident in the images (Figure 9d,e) that G–GO has a smooth and uniform surface with typical wrinkle morphology, and presents a lamellar structure according to the cross-section image. Thus, the SEM images of the initial materials, BAC and G, and the materials obtained from them using the Hummers’ method, BAC–GO and G–GO, have distinctive structural features, peculiar morphological features, and, according to the pictures, it is clearly seen that they have been successfully synthesized. Therefore, they are in good agreement with the UV spectra, Raman spectroscopy, IR, XPS, XRD, EDX (Figures 4–11), and determination of the level of oxygen-containing groups (Table 2), as well as the known literature data [11,14,40,50,72,73,74,75,76,77,78,79,80,81,82,83,84,85,86,87,96,97,98,99,100].

### 3.8. EDX Analysis

We have obtained EDX analysis of the samples of G, BAC, G–GO, and BAC–GO, which have peculiar, distinctive features depending on the initial materials, G and BAC. According to the results of the EDX analysis and the C/O values, one can observe how the atomic fraction of oxygen increases after the oxidation of G and BAC using the Hummers’ method.

**Figure 12:**
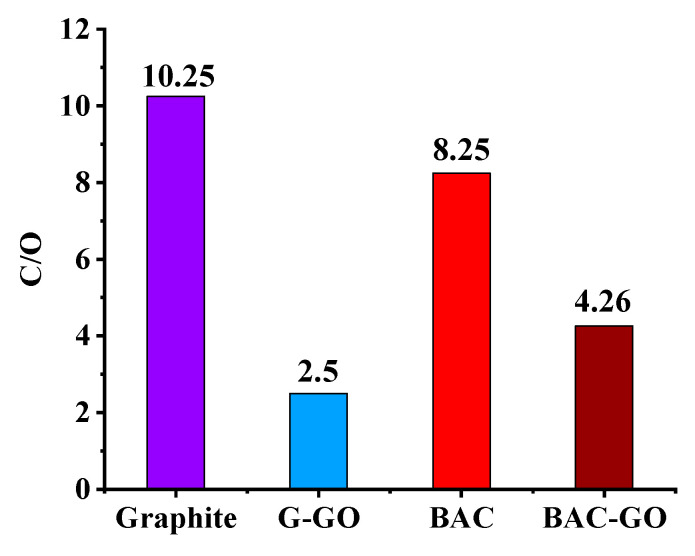
C/O ratio of graphite, G–GO, BAC, and BAC–GO.

Elemental analysis showed (Figure 11 and Figure 12) that each sample contained the following different percentages of C and O and C/O ratios: initial sample G contained C—91.11 at.%; O—8.89 at.% (C/O = 10.25), and from it, the obtained G–GO has C—71.41 at.%; O—28.59 at.% (C/O = 2.50). Respectively, BAC contains C—89.19 at.%; O—10.81 at.% (C/O = 8.25), and synthesized BAC–GO from it has C—81.02 at.%; O—18.98 at.% (C/O = 4.26). In the Figure 11 and Figure 12, and according to the obtained values, it is clearly seen that the values of the C/O ratio decrease with an increase in the atomic fraction of oxygen; the atomic fraction of oxygen before the oxidation of graphite was 8.89 at.%, and after, it became 28.59 at.%, which is an almost 3.32 times increase. The same difference is observed in the BAC and BAC–GO samples, where the O content was 10.81 at.%, and after oxidation, it became 18.98 at.%, which is an increase of almost 1.7 times. The value of the atomic fraction of oxygen of the original BAC compared to the original graphite is almost 1.2-times higher, which is due to the sorption of oxygen from the air atmosphere, due to the more highly porous surface of BAC compared to graphite.

However, a comparison of the EDX results of the obtained BAC–GO and G–GO samples showed that the amount of the atomic fraction of oxygen in G–GO is almost 1.5-times higher than in BAC–GO. This is in good agreement with the UV spectra, Raman spectroscopy, IR, XPS, XRD, SEM (Figures 4–11), and determination of the level of oxygen-containing groups (Table 2) [11,14,50,72,73,81,82,84,85,86,87,96,97,98,99,100]. Compared to G–GO, the low amount of oxygen atomic fraction in BAC–GO is presumably because in the G–GO sample, a large number of functional oxygen-containing groups are attached on both sides of the basal plane and along the edges. While in BAC–GO, the oxygen groups presumably penetrate into tunnel-shaped pores, and, due to the lack of a plane and a disordered structure, they adhere along the peripheries [11,13,21,23,30,40,56,74,75,76,77,78,79,80,83].

### 3.9. Brillouin Spectra (Elasticity and Viscosity), Density, Sound Velocity, and Young Modulus Results

Results from this experimental part are presented in Table 7. According to the obtained results, one can see the distinctive features between G–GO and BAC–GO depending on the initial materials. The elasticity value for BAC–GO was 7.47, and for G–GO it was equal to 7.55, which is more than in BAC–GO, and the viscosity values for the BAC–GO sample turned out to be 0.68, which is slightly more than for G –GO which was equal to 0.67. Changes in the values of elasticity and viscosity are in good agreement with the density, sound velocity, and Young’s modulus of the samples of G–GO and BAC–GO. In the density values according to Table 7, there is also a low reading for BAC–GO as it was for the results of elasticity; respectively, the density of BAC–GO is 0.99 g/cm^3^, and G–GO is 1 g/cm^3^, which is more than BAC–GO. Similar changes are seen in the sound velocity and viscosity results of BAC–GO, in which the sound velocity of BAC–GO was 1483.91 m/s, slightly more than G–GO, which is 1483.83 m/s. The changes in the results of the Young’s modulus are similar to the values of the speed of sound, for which the BAC–GO value (2.17 GPa) is also more than in G–GO (2.2 GPa).

In general, the change in the results of Brillouin (elasticity and viscosity), density, and Young’s modulus of the G–GO and BAC–GO samples was facilitated by the structure of the initial materials, as well as the approximate arrangement of functional oxygen-containing groups. Due to the lamellar shape of the aromatic nature of the ordered structure in graphite, oxygen groups are seated on the basal plane and along the edges. While in BAC, these groups are seated along the periphery and inside the tunnel pores due to the rod-shaped porous form of the aliphatic nature of the saturated carbon bonds [11,13,21,23,30,40,56,74,75,76,77,78,79,80,83]. Thus, the obtained values are in good agreement with the previous results of UV, Raman spectroscopy, IR, XPS, XRD, SEM, EDX (Figures 4–11), and determination of the level of oxygen-containing groups (Table 2) [11,14,50,72,73,81,82,84,85,86,87,96,97,98,99,100], which confirms successful receipt of G–GO and BAC–GO.

Young’s modulus was calculated according to the following formula:

E = pV^2^ (4)

where

p—density;
V—sound velocity.

### 3.10. Electrical Characteristic

**Figure 13:**
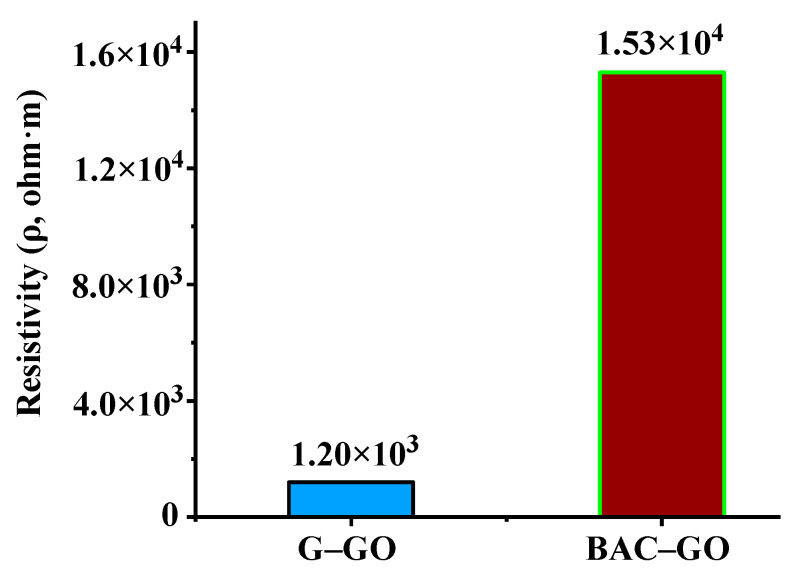
Electrical characteristics of graphite, G–GO, BAC, and BAC–GO.

The electrical characteristics of the G–GO and BAC–GO samples were measured using the Keithley picoammeter (Model 6485) with four-point probes. The resistivity results showed the following values: ρ (G–GO) = 1.2 × 10^3^ and ρ (BAC–GO) = 1.53 × 10^4^ Ohm × m (Figure 13). According to the obtained resistivity values, there is a difference between G–GO and BAC–GO; for the BAC–GO sample, ρ has become an order of magnitude greater than for G–GO. The high value of BAC–GO resistivity is due to the fact that BAC crystals by their nature have a random porous rod-like structure of the aliphatic character of saturated carbon bonds (Figure 9a,c) [11,13,23,35,40,56,59,60,61,62,63,64,65,66,67,68,69,70,71,74,75,76,77,78,79,80,83,101]; respectively, the conductivity of the electric current is lower than that of G–GO. Graphite crystals mainly have flocculent, scaly, or lamellar forms of an aromatic nature of the ordered structure [11,35,74,75,76,77,78,79,80,83,101] (Figure 9b,d), which allows for good current conductivity and shows a lower value of resistivity than in BAC. In addition, the high value of BAC resistivity is presumably influenced by the location of functional oxygen-containing groups, along the periphery and inside the tunnel rod-like porous form of BAC (Figure 9a,c) [11,14,35,72,73,74,75,76,77,78,79,80,81,82,83,84,96,101], as well as the presence defective surface (Figure 6) (Table 1) [21,54,55,56,59,88,89,90,91,92,93,94,95]. This is in good agreement with the results of UV, XPS, IR, Raman, XRD, SEM, EDX (Figures 4–11), and determination of the level of oxygen-containing groups (Table 2).

### 3.11. Adsorption Porosimetry

**Figure 14:**
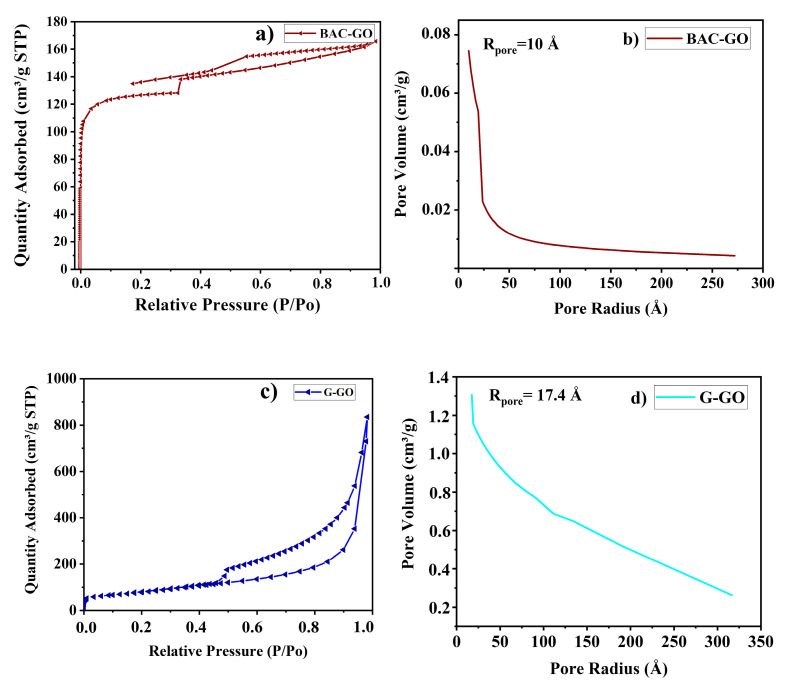
Nitrogen adsorption–desorption isotherm and pore sizes of (**a**,**b**)—BAC–GO and (**c**,**d**)—G–GO.

Figure 14 illustrates the nitrogen absorption–desorption study of BAC–GO and G–GO. According to the research on BAC–GO, the P/Po range was between 0.1 and 1.0 [50]. The total BET surface area of BAC–GO is 496.95 m^2^/g, and the average pore size distribution, calculated using the BJH method from the nitrogen desorption curve, is 10 Å. This is a result of the capillary condensation of nitrogen in the mesopores of GO. This type of hysteresis corresponds to a type III isotherm, indicative of an unlimited adsorption–desorption process occurring at P/Po [50,102].

In the nitrogen sorption–desorption isotherm of G–GO, the total BET surface area is 252.98 m^2^/g, and the pore size distribution, calculated using the BJH method from the nitrogen desorption curve, shows an average pore size of 17.4 Å. The observed hysteresis follows the general form of type H3 [103]. It was found that the active surface area of GO obtained from activated carbon is 1.96-times higher than that of GO obtained from graphite. This is attributed to the large specific surface area of the initial activated carbon. The pore radius of BAC–GO was found to be 7.4 Å smaller than that of G–GO. This is due to the morphological characteristics of the raw materials. The surface morphology of primary activated carbon is mesoporous, and graphite is characterized by a sheet-like sandwich structure, which may be due to the absence of pores [104,105].

### 3.12. Electrophysical Characteristics of the Humidity Sensor Based on BAC–GO and G–GO

**Figure 15:**
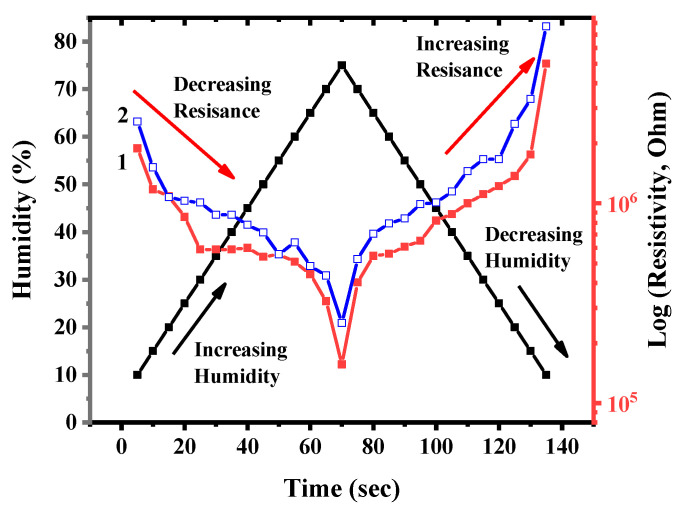
Dynamics of response and recovery of (1) *BAC–GO* and (2) *G–GO*.

The electrical parameters of the humidity sensor based on the BAC–GO and G–GO samples were studied as a function of humidity to determine the performance of the device. The response and recovery of the humidity sensor were tested using a Keithley (Model 6485) meter attached to copper electrodes with alligator clips. The results of the sensor response and recovery tests as a function of time are shown in Figure 15. The response and recovery time of the humidity sensor was tested in a sealed chamber with a controlled humidity level in the range from 10% to 75% (RH) with an interval of 5% (RH) for 135 s (Figure 15). The results of this study showed that with an increase in the relative humidity, the electrical resistance of the humidity sensor decreased.

For the G–GO sample, with an increase in humidity from 10% to 75% (RH), the electrical resistance decreased by three orders of magnitude from 1.8 × 10^6^ to 1.5 × 10^5^ Log (R, Ohm), and in the BAC–GO sample, the electrical resistance decreased—(from 2.5 × 10^6^ up to 2.5 × 10^5^) Log (R, Ohm). Accordingly, in the dynamics of recovery, with a decrease in humidity from 75% to 10%, the electrical resistance for G–GO and BAC–GO samples increased in the following order: G–GO—(from 1.5 × 10^5^ to 5 × 10^6^); BAC–GO—(from 2.5 × 10^5^ to 7.6 × 10^6^) Log (R, Ohm). In general, during the response of the sensor, the process of reducing the electrical resistance with increasing humidity is due to the fact that a large number of water molecules are adsorbed on the surface of BAC–GO and G–GO, which significantly increases its conductivity. Accordingly, during the dynamics of recovery, adsorbed water molecules are removed, which leads to an increase in resistance. Thus, according to the results of the dynamics of response and recovery of the humidity sensor, a decrease and increase in the electrical resistance values is observed in the entire range of relative humidity, which confirms the sensitivity of BAC–GO and G–GO membranes to humidity.

## 4. Conclusions

We have obtained GO from a renewable natural raw material of plant biomass, BAC, which is an economically efficient, safe, and suitable starting material for large-scale production of graphene-like nanostructures. We have demonstrated the results of a study of the similarity of the physicochemical structure of BAC–GO and G–GO, as well as presented unique differences in surface morphology, and electrical, and mechanical characteristics. The morphological properties of BAC–GO and G–GO showed a difference depending on the initial material, BAC and graphite; undoubtedly, the nature of the materials, with BAC having a rod-like tunnel porous structure and graphite having a lamellar flake-like structure, respectively, affected the location and content of functional oxygen-containing groups and physicochemical and electrical properties. Consequently, the results of EDX analyses of BAC–GO confirmed the content of C—89.19 at.%—and O—10.81 at.% (C/O = 8.25)—which is slightly less than in G–GO which showed C of 81.02 at.% and O of18.98 at.% (C/O=4.26). Also, successful oxidation of BAC was proven via XPS analyses, where the main peaks were found with binding energies of 284.1 eV and 288.8 eV. These are associated with aromatic/aliphatic carbon atoms sp^2^/sp^3^ (C=C), epoxy groups (C-O-C), and carboxyl groups (C(O)-O-H), respectively, and peaks in the region of 292–295 eV, which are characteristic of the binding energies of π-π* transitions in aromatic closed chains. G–GO peak position data were displayed at 284.6; 286.6; and 288.8 eV. In addition, the successful preparation of BAC–GO was confirmed with the results obtained via FTIR, Raman spectroscopy, and XRD. In the FTIR spectrum, after strong oxidation of BAC and graphite, the appearance of functional oxygen-containing groups associated with carbon atoms was found; therefore, it affected the increase in the interplanar distance by almost 2.28 times (from 3.59 to 8.2 Å). In addition, an increase in the Raman I_D_/I_G_ intensity ratio by almost 1.12 times (from 1.23 to 1.38) compared to the initial BAC was observed, which approximately corresponded with G–GO. The same changes were found in the results of Brillouin (elasticity and viscosity), density, Young’s modulus of G–GO and BAC–GO samples, where, in terms of elasticity and density, G–GO showed almost 1.01-times higher values than BAC–GO. Moreover, according to the values of viscosity, sound velocity and Young’s modulus, the BAC–GO sample turned out to be slightly higher by 1.01 times than that of G–GO. According to the results of the electrical characteristic, the resistivity value ρ (BAC–GO) of 1.53 × 10^4^ Ohm × m is almost an order of magnitude higher than ρ (G–GO) = 1.2 × 10^3^ Ohm × m. In general, our results showed successful oxidation of BAC via the Hummers’ method and, therefore, are in good agreement with the well-known literature data, and the distinctive structural and physicochemical features and unique differences between G–GO and BAC–GO depend mainly on the nature of the initial materials of graphite and BAC. Thus, we note that the production of BAC–GO from renewable natural raw materials is a potential cost-effective material for the large-scale production of graphene nanostructures, which, in turn, will expand the fields of application in nanoelectronics, medicine, etc. 

## Figures and Tables

**Table 1:** Functional groups of G–GO and BAC–GO according to UV and FTIR spectra.

Samples	Functional Groups								
	UV Spectra, λ, Wavelength (nm)		FTIR Spectra, Wavenumber (cm^−1^)						
	228	233	980	1054	1249	1585	1723	1420	3300
**G–GO**	presence of π-π* bond of aromatic ring in GO molecule and C=O bonds in epoxy and carbonyl groups		C-O-H and C=O bonds	C–O alkoxy bonds	C–O epoxy functional groups	C=C bonds of the aromatic ring	C=O bonds in carbonyl group and carboxyl groups	O-H	
**BAC–GO**									

**Table 2:** Total number of oxygen-containing groups in carbon materials.

Samples	Total Number of Oxygen-Containing Groups, mg-eq/g
BAC	0.2 ± 0.01
Graphite	0.2 ± 0.01
BAC–GO	13 ± 0.2
G–GO	15 ± 0.2

**Table 3:** Ratios of the D and G bands (I_D_/I_G_) and crystal sizes of samples.

Samples	2D	D-Band Position (cm^−1^)	G-Band Position (cm^−1^)	I_D_ (arb.u.)	I_G_ (arb.u.)	L_a_ (nm)	I_D_/I_G_
G	2747	1360	1582	0.3642	1	33.09	0.3642
G–GO	Broad peak 2600–2800	1357	1589	0.8813	1	13.69	0.88
BAC		1360	1595	1	0.8148	9.8	1.23
BAC–GO		1354	1598	1	0.7158	8.67	1.39

**Table 4:** Functional groups of G–GO and BAC–GO according to UV, FTIR, and XPS spectra.

Samples	Functional Groups										
	FTIR Spectra, Wavelength (cm^−1^)							XPS Spectra Binding Energy (E) eV			
								C1s		O1s	
	980	1054	1249	1585	1723	1420	3300	284.6; 286.6 288.8	284.1; 288.8; 292–295	532.5	532.6
**G–GO**	C-O-H and C=O bonds	C–O alkoxy bonds	C–O epoxy functional groups	C=C bonds of the aromatic ring	C=O bonds in carbonyl group and carboxyl groups	O-H		sp^2^ aromatic carbon atoms (C=C), epoxy groups (C-O-C), and carboxyl groups (C(O)-O-H)		C=O C–O ketone, carbonyl, hydroxyl, and epoxy functionalities	
**BAC–GO**									sp^2^ carbon (C-C/C=C, 284.1 eV); carbonyl/carboxyl groups (C=O/O-C=O, 288.8 eV); π-π* transitions in aromatic closed chains in the region of 292–295 eV		

**Table 5:** XRD of G–GO from G and BAC–GO from BAC.

Samples	d Inter-Layer Distance (Å)
**BAC**	3.59
**Graphite**	3.5
**BAC–GO**	8.2
**G–GO**	10.2

**Table 6:** Crystallite sizes of G–GO from G and BAC–GO from BAC.

Samples	Parameters		Peak Position	θ (°) (2θ(°)/2)	β		cos θ(°)	Crystallite Size (Å) D	Crystallite Size (nm) D
	K [37]	Λ (Å)	2θ(°)		FWHM (In Degree)	FWHM (In Radian)			
graphite	0.94	1.5418	26.43	13.21	0.55	0.009595986	0.79	120.31	12.03
G–GO	0.94	1.5418	7.05	3.53	0.27	0.004807155	0.92	279.33	27.93
BAC	0.94	1.5418	23.46	11.73	7.63	0.13320925	0.67	7.30	0.73
BAC–GO	0.94	1.5418	11.91	5.96	1.48	0.025833508	0.95	53.15	5.31

**Table 7:** Brillouin Spectra (Elasticity and Viscosity), Density, Sound Velocity, and Young Modulus Results.

Samples	Brillouin Shift (Ghz) Elasticity	Brillouin Line Width Viscosity	Density (g/cm^3^)	Sound Velocity (m/s)	Young Modulus GPa
BAC–GO	7.47	0.68	0.99	1483.91	2.17
G–GO	7.55	0.67	1	1483.83	2.2

## Data Availability

The datasets generated during and/or analyzed during the current study are available from the corresponding author on reasonable request.
